# Nanostructured ZnFe_2_O_4_: An Exotic Energy Material

**DOI:** 10.3390/nano11051286

**Published:** 2021-05-13

**Authors:** Murtaza Bohra, Vidya Alman, Rémi Arras

**Affiliations:** 1 Department of Physics, École Centrale School of Engineering (MEC), Mahindra University, Survey Number 62/1A, Bahadurpally Jeedimetla, Hyderabad 500043, India; vidyavasant20pphy010@mahindrauniversity.edu.in; 2 Centre d’Elaboration de Matériaux et d’Etudes Structurales (CEMES), Université de Toulouse, CNRS, UPS, 29 rue Jeanne Marvig, F-31055 Toulouse, France; remi.arras@cemes.fr

**Keywords:** inverted ZnFe_2_O_4_, nanostructuration, energy harvesting and storage

## Abstract

More people, more cities; the energy demand increases in consequence and much of that will rely on next-generation smart materials. Zn-ferrites (ZnFe_2_O_4_) are nonconventional ceramic materials on account of their unique properties, such as chemical and thermal stability and the reduced toxicity of Zn over other metals. Furthermore, the remarkable cation inversion behavior in nanostructured ZnFe_2_O_4_ extensively cast-off in the high-density magnetic data storage, 5G mobile communication, energy storage devices like Li-ion batteries, supercapacitors, and water splitting for hydrogen production, among others. Here, we review how aforesaid properties can be easily tuned in various ZnFe_2_O_4_ nanostructures depending on the choice, amount, and oxidation state of metal ions, the specific features of cation arrangement in the crystal lattice and the processing route used for the fabrication.

## 1. Introduction

Regardless of the environmental pressure to reduce energy consumption, global power demand is growing—and one of the ways to solve this “looming energy crisis” is through the exploration of novel earth-abundant energy materials [1,2,3,4]. Further, the pace of technological change is getting faster, thus the miniaturization of electronic devices is also key [5,6,7]. These demands can be met by realizing an efficient oxide energy material at the nanoscale by controlling their complex crystal structure with many degrees of freedom (i.e., charge, spin, and orbital) [8]. Oxide materials usually possess high density, display robust physical properties, and show great flexibility to tune their optical, electrical, and magnetic properties, with subtle changes such as elemental substitutions, defect, and strain engineering [1,2,3,4,5,6,7,8]. Among the oxides, earth-abundant Zn-ferrite can be the potential alternative energy material. Zn-ferrites exhibit a unique set of functional properties [9,10,11,12,13,14,15,16,17,18,19,20,21]; it possesses a normal spinel structure (ZnFe_2_O_4_) in the bulk form at room temperature, whereas inverted spinel structure has been observed at the nanoscale. The electronic band structure calculations predict the insulating character of ZnFe_2_O_4_ [9,10,11,12]. The reported room temperature resistivity value of ZnFe_2_O_4_ is *ρ* = 2 × 10^2^ (Ω·cm), which is two to three orders of magnitude lower than other spinel ferrites [13]. The optical band gap energy [14,15], with values of 2.02 eV and 2.33 eV for the indirect and direct transition near the maxima of UV-Visible absorption curves is suitable for energy harvesting with sufficient amounts of electron-hole pair generation from the solar spectrum, and suitable to drive redox reactions with proper band positions [16]. Appropriate doping of Zn in Fe_3_O_4_ [17], i.e., a good control of the Zn*_y_*Fe_3−*y*_O_4_ stoichiometry, is one way to tune electric, magnetic, and optical properties which provides a versatile playground to build ZnFe_2_O_4_ based sensors, solid-state energy conversion devices, and solar cells. The nanostructuration of ZnFe_2_O_4_ is a second lever of action which enables external tuning competence of the properties by inducing the cation inversion [18,19,20]. The cation inversion, an interchange of Zn and Fe atomic positions in the spinel ZnFe_2_O_4_ strongly depends upon the size of nanostructures produced by different growth methods [19,21]; varying the extent of cation-inversion in spinel structures allows tuning their electronic and spin structure, which eventually can be used to design various spintronic, microwave, and photoelectrochemical (PEC) functionalities. The most important cost factor in energy storage applications is light-absorbing material’s efficiency. In the case of ZnFe_2_O_4_, the theoretical solar-to-hydrogen (STH) conversion efficiency is estimated to be 17.9% for PEC water splitting [22]. Furthermore, the morphology and size of ZnFe_2_O_4_ as electrode material are crucial factors in supercapacitors and lithium-ion batteries, wherein theoretical capacity for supercapacitor (2600 F/g) and Li-ion battery (1072 mAhg^−1^) are higher than when using other ferrites [23,24]. The synergies between Zn and Fe ions in Fe-based binary oxides with large surface area offers higher electrochemical kinetics, active sites, and delivers superior capacitance [25]. Thus, the ability to strongly tune the overall properties of nanostructured ZnFe_2_O_4_ material demonstrates its pertinence in synergist energy storage applications as well. 

Ever since then, ZnFe_2_O_4_ has been an object of extensive research from both experimental and theoretical points of view, due to its modified and unusual properties that emerge at the nanoscale. In this review, we will emphasize how nanostructured ZnFe_2_O_4_ oxide can convert, harvest, store, or produce energy. Despite the progress in mastering the nanostructuration of ZnFe_2_O_4_, there are open questions that are yet to be fully understood. We will shed light on some of these questions such as ZnFe_2_O_4_-property modification at the nanoscale, sensitivity to oxygen stoichiometry, particle size, and surface morphology effects. Finally, we will discuss how ZnFe_2_O_4_ nanostructures are currently being employed in supercapacitors, lithium-ion batteries, water splitting, low-energy-consumption spintronic, and microwave technologies, which would ultimately offer guidelines for designing futuristic energy-efficient devices.

## 2. Material Properties of ZnFe_2_O_4_

### 2.1. Bulk Crystalline and Spin Structure

**Figure 1:**
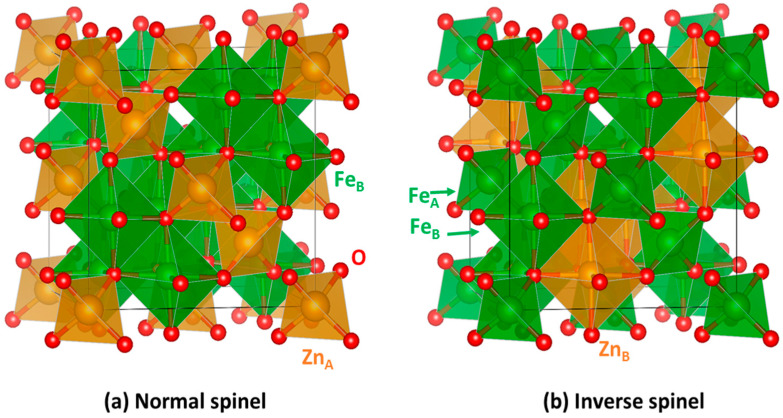
Atomic structure of the spinel zinc ferrite with (**a**) normal and (**b**) inverse cation distributions. In each case, the conventional cubic cell (with 8 f.u. of ZnFe_2_O_4_) is delimited by solid black lines. Tetrahedral (A) and Octahedral (B) atomic coordination environments can also be identified by their polyhedra. Orange, green, and red atoms correspond to Zn, Fe and O chemical elements, respectively.

Bulk ZnFe_2_O_4_ possesses a normal cubic spinel XY_2_O_4_ structure (space group 227-*Fd*$\bar{3}$*m*; *a* = 8.44 Å) (Figure 1a), wherein oxygen anions occupy 32*e* Wyckoff sites and form a distorted face-centered cubic (FCC) lattice and large interstices between O^2−^ are partially occupied by iron and zinc cations. One eighth of the tetrahedral positions (labelled A, Wyckoff positions 8*a*) are occupied by divalent Zn^2+^ cations, while half of the octahedral positions (labelled B, Wyckoff positions 16*d*) are occupied by trivalent Fe^3+^ cations, leading to the formula [Zn^2+^]_A_[Fe^3+^_2_]_B_O^2−^_4_ [26]. One of the distinctive features of the spinels, however, is the wide range of cation distributions accessible in this system and not all spinels have the normal structure as their ground state configuration. There exist several chemistries with the “inverse” spinel configuration where the tetrahedral sites are occupied by the trivalent Y atoms and the octahedral sites are shared equally by both the divalent, X, and trivalent, Y, atoms, i.e., [Y]_A_[XY]_B_O_4_ (Figure 1b) [27]. At a finite temperature, mixing of elemental species within the octahedral lattice or across the octahedral and tetrahedral lattices is often observed; it is then possible to define the inversion degree *x* of the spinel leading associated to the following cation distribution: [X_1−*x*_Y*_x_*]_A_[X*_x_*Y_2−*x*_]_B_O_4_. The inversion parameter can vary from 0 (for a normal spinel) to 1 (for an inverse spinel) and adopts a value of 2/3 for a completely random distribution of the metal atoms [28].

**Figure 2:**
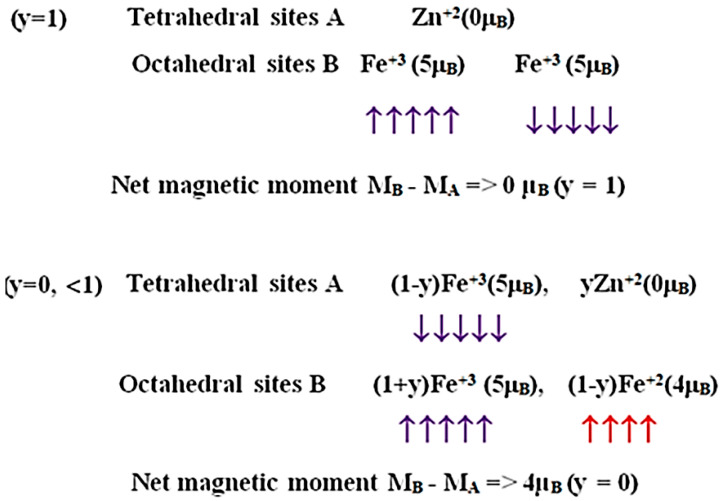
The spin magnetic moment per formula unit of Zn_y_Fe_3−y_O_4_ for *y* = 0 and 1.

Bulk normal spinel ZnFe_2_O_4_ ideally contains only one type of magnetic ions (Fe^3+^) and possesses the following magnetic structure (Figure 2). In conventional spinel ferrites, we can mostly consider two superexchange interactions *J*_BB_, and *J*_AB_ between the magnetic ions on the A and B sites and mediated by the oxygen ions. According to the Goodenough–Kanamori–Anderson rules [29,30], the dominant exchange interaction is *J*_AB_ and corresponds to an antiferromagnetic coupling between atoms in tetrahedral and octahedral sites, because of the nearly 125° angle formed by the oxygen bridge linking these two sites; it turns out to be the ferrimagnetic ordering in many spinel ferrites. However, as in the normal spinel ZnFe_2_O_4_, the tetrahedral sites are occupied by diamagnetic Zn^2+^ cations (*M*(Zn^2+^) = 0 μ_B_), such interaction is absent and only a weak antiferromagnetic interaction *J*_BB_ is operative between Fe^3+^ ions in octahedral sites [19]. This causes the Néel temperature of this oxide to be very low, around 10 K, ZnFe_2_O_4_ being paramagnetic at room temperature.

**Figure 3:**
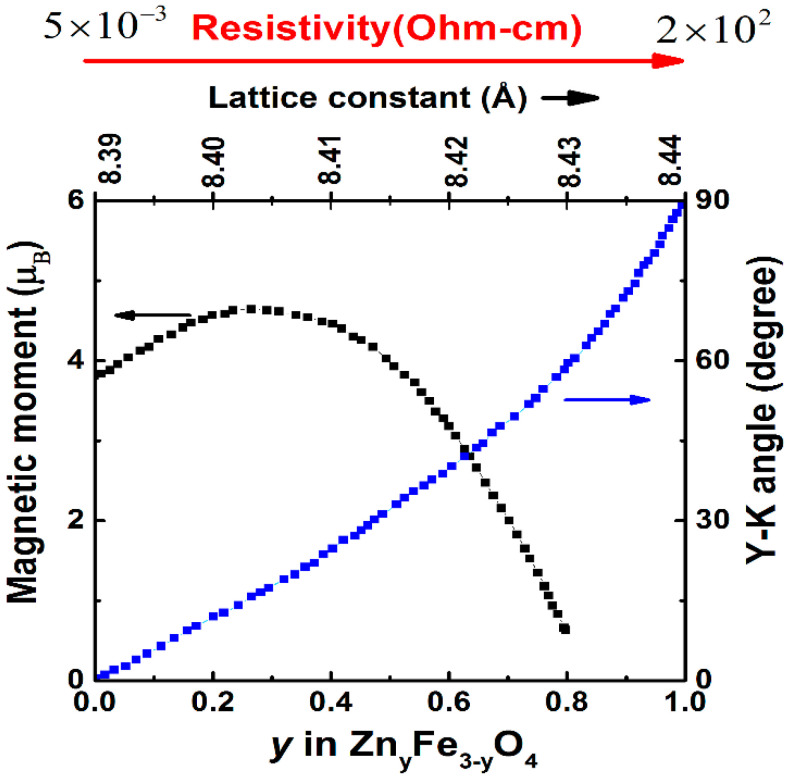
Magnetic moment, Yafet–Kittel angles, and resistivity as a function of *y* in Zn_y_Fe_3−y_O_4_ at 300 K (Reproduced with permission from [17]. Copyright American Physical Society, 1976).

A higher Néel temperature can be obtained in the Zn*_y_*Fe_3−*y*_O_4_ compound above room temperatures by varying Zn doping concentration (*y*) in Fe_3_O_4_ [17], as shown in Figure 3. The spin structures can be given as mixed-valence [(Zn^2+^)*_y_*(Fe^3+^)_1−*y*_]_A_[(Fe^3+^)_1+*y*_(Fe^2+^)_1−*y*_]_B_(O^2−^)_4_ inverse spinel, as shown in Figure 2. At low *y*, the magnetic moments of the A sites are antiparallel to those of the B sites, so the net magnetic moment of the Zn*_y_*Fe_3−*y*_O_4_ is *M*_S_ = *M*_B_ − *M*_A_ = (4 + 6*y*) μ_B_. For *y* < 0.25, the *M*_S_ of Zn*_y_*Fe_3−*y*_O_4_ increases with increasing *y*, see Figure 3. However, at high Zn contents, the total magnetization is expressed by *M*_S_ = *M*_B_ cos*α*_YK_ − *M*_A_, where *α*_YK_ is the Yafet–Kittel canting angle [17,31,32] between the magnetic moments in the B sites. For *y* > 0.25, the magnetic moments of remaining Fe^3+^ ions located in the A sites are no longer able to force an antiparallel alignment to all the moments of the iron ions in the B sites. The B sites will then divide themselves into sublattices and the associated magnetic moments will rotate, forming a canting angle between each other, and in consequence of which, a further replacement of the Fe^3+^ ions by the Zn^2+^ ions will lead to a decrease of the magnetic moments in the B sites, that is a decrease of the total *M*_S_. 

**Figure 4:**
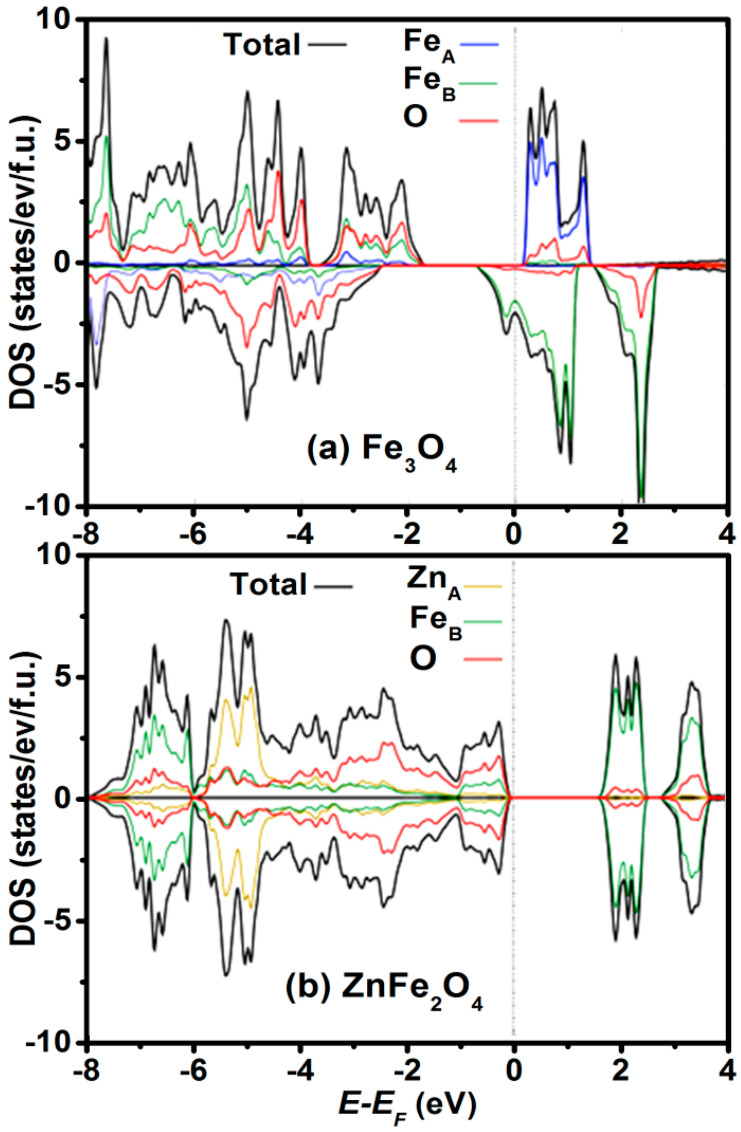
Spin-projected densities of states (DOS) of the Zn_y_Fe_3−y_O_4_ bulk compound obtained from the DFT calculations, using a GGA+*U* (*U*(Fe,3d) = 4.0 eV) approximation. (**a**) *y* = 0 corresponds to the half-metallic and ferrimagnetic magnetite Fe_3_O_4_ and (**b**) *y* = 1 to the insulating and antiferromagnetic ZnFe_2_O_4_. Positive and negative DOS represent, respectively, the projection onto majority and minority spin states.

We now discuss the relationship between electronic structures and the physical properties of the Zn*_y_*Fe_3−*y*_O_4_ system. With increasing Zn doping, the cubic unit cell expands from 8.39 Å to 8.44 Å, but at the same time induces a decrease of [Fe^2+^]_B_/[Fe^3+^]_B_ ratio, which in turn reduces the number of itinerant electrons in *t*_2*g*_ states at Fermi level, as shown in Figure 4 [33,34,35,36,37]. This causes change in electric resistivity from 5 × 10^−3^ Ω·cm of Fe_3_O_4_ to 2 × 10^2^ Ω·cm for ZnFe_2_O_4_, opening several applications where tunable electric and optical properties are required. However, because of preparation difficulties (particularly in controlling the Fe^2+^/Fe^3+^ ratio), these Zn*_y_*Fe_3−*y*_O_4_ compounds have not been so far commercially exploited. 

### 2.2. Cation Engineering in Nano Regime

Several methods, such as heat treatments, chemical replacements, and the nanostructuration of materials, have been employed to manipulate the distribution of cations between the two interstitial sites of the spinel structures, controlling their physical behaviors [38,39,40]. Among these methods, stoichiometric ZnFe_2_O_4_ show striking changes in its crystalline properties by reducing the grain size to the nanometer-sized range [21,40]. When it is prepared at the nanoscale, the energy associated with the low particle size favors a mixed cation distribution in which the Zn^2+^ and Fe^3+^ ions are distributed along the A and B sites giving rise to the inverse spinel structure;

[(Zn^2+^)_1−*x*_(Fe^3+^)*_x_*]_A_[(Zn^2+^)*_x_*(Fe^3+^)_2−*x*_]_B_(O^2−^)_4_

The size of the nanostructures and the resulting cation inversion degree (*x* = 0 to 0.94) vary in different reports, as stoichiometry commonly depends on growth conditions [41]. The inverted ZnFe_2_O_4_ nanostructures have many applications in spintronic and microwave devices and water splitting. These ZnFe_2_O_4_ nanostructures with tunable surface morphology and electrical conductivity are further useful in Li-ion battery and supercapacitors as an electrode [23,24]. Thus, simultaneous measurement of saturation magnetization and conductivity can be used as a tool to approximate cation inversion in the ZnFe_2_O_4_ compound. However, in an oxygen-deficient growth regime, the likely inverted spinel structure is [42,43]

[(Zn^2+^)_1−x−y_(Fe^3+^)_x+y_]_A_[(Zn^2+^)*_x_*(Fe^2+^)*_y_*(Fe^3+^)_2−x−y_]_B_(O^2−^)_4−δ_

wherein partial reduction of Fe^+3^ into Fe^+2^ is expected at B sites. For a higher oxygen-deficient growth regime, now there is no Zn^+2^ ions are available at B sites and inverted spinel structure is represented a [17]

[(Zn^2+^)*_x_*(Fe^3+^)_1−x_]_A_[(Fe^2+^)_1−x_(Fe^3+^)_1+x_]_B_(O^2−^)_4−δ_

akin to the Zn doped Fe_3_O_4_ (Zn*_y_*Fe_3__−*y*_O_4_) compound. In extreme condition (high temperature and low oxygen pressure), there is a high chance of dissociation of ZnFe_2_O_4_ into Fe_3_O_4_ and ZnO compounds [44]. These transformations greatly affect the magnetic, electric, and optical properties of ZnFe_2_O_4_ nanostructures.

## 3. Various ZnFe_2_O_4_ Nanostructure Morphologies

The physical properties of nanostructured ZnFe_2_O_4_ can be easily tuned depending on the choice, amount, and oxidation state of metal ions, depending on specific features of cation arrangement in the crystal lattice and finally, depending on the processing route. Recently, a lot of work has been carried out on various nanostructured ZnFe_2_O_4_ morphologies, including single crystals, epitaxial and polycrystalline thin films, nanoparticles, arrays of colloidal nanocrystals, and heterostructures. The eventual utilization of these morphologies is to develop novel storage devices and this is a critical issue for further investigations. 

### 3.1. Nanoparticles (1 nm < Particle Size < 100 nm)

**Figure 5:**
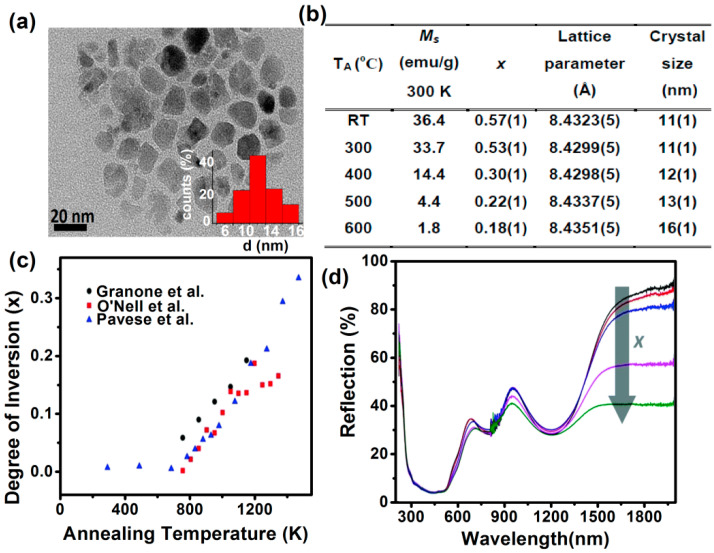
(**a**) Low-magnification TEM image of ZnFe_2_O_4_ nanoparticles of average size 11 nm. (**b**) Cation inversion and lattice parameter, Ms and crystalize at different annealing temperatures (Reproduced with permission from [40]. Copyright American Chemical Society, 2019). (**c**) Degree of inversion, *x*, versus the annealing temperature comparison of result with values obtained by different groups (Reproduced with permission from [42]. Copyright PCCP Owner Societies, 2018). (**d**) UV-diffuse reflectance spectrum of ZnFe_2_O_4_ nanoparticles with increasing degree of inversion (— *x* = 0.074; 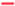 *x* = 0.104; 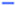 *x* = 0.134; 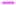 *x* = 0.159;  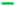 *x* = 0.203).

The recent advances in the synthesis of uniform and size-controllable ZnFe_2_O_4_ nanocrystals have enabled the size-dependent characterization of their physical properties, as well as their use in applications. The cation inversion in ZnFe_2_O_4_ nanoparticles varies from method to method. At standard conditions (273 K and 100 kPa), the normal structure is the thermodynamically most stable configuration for bulk ZnFe_2_O_4_, however, nanosized ZnFe_2_O_4_ exhibits a partially inverted spinel structure, which further undergoes structural changes from orthorhombic (30 GPa) to tetragonal (55 GPa) structure at high pressures [28]. Cobos, et al. [40] explored the relationship between microstructure and magnetic properties of ZnFe_2_O_4_ nanoparticles. The high inversion degree (*x* ≈ 0.6) is obtained after 150 h milling with a size of 11 nm in as-milled samples, as shown in Figure 5a, and afterwards, the inversion degree is modified by thermal treatments at different temperatures, i.e., 300, 400, 500, and 600 °C, to finally obtain a very low inversion degree (*x* ≈ 0.15), as reported in Figure 5b. It can be noticed that even though the degree of inversion has changed significantly, there is hardly any effect on lattice parameters (8.43–8.44 Å). For *x* < 0.25, antiferromagnetism, ferrimagnetism, and spin frustration can coexist; however, pure ferrimagnetic phase with a maximum magnetization (*M*_S_ = 3.5μ_B_ per formula unit) can be obtained for 0.25 < *x* < 0.5. Finally, for *x* > 0.5, a new antiferromagnetic order appeared due to the overpopulation of nonmagnetic Zn on octahedral sites that leads to equally distributed magnetic cations in octahedral and tetrahedral sites.

A comprehensive work regarding the thermodynamics of the cation disorder and the dependence of the degree of inversion with the temperature has been done by many groups (Figure 5c). The cation distribution in pure ZnFe_2_O_4_ can be treated as a dynamic equilibrium according to the following interchange reaction, Zn_A_ + Fe_B_ ⇄ Zn_A_ + Fe_A_ between ions being present in A and B sites. Despite we know that the distribution of cations (Fe^3+^ and Zn^2+^) in an inverse spinel structure governs physical properties, its effect other than magnetic properties of ZnFe_2_O_4_ are not deliberately explored. Five ZnFe_2_O_4_ samples with degrees of inversion varying from 0.07 to 0.20 were prepared using a solid-state reaction by Granone, et al. [42] with different annealing temperatures and subsequent quenching. However, their optical band gap energy, with values of 2.02 eV and 2.33 eV for the indirect and direct transition, respectively, is found to be independent of the degree of inversion, as can be seen in the UV-Vis-NIR spectra in Figure 5d. No effect on transition energies due to ligand (O^2−^)-to-metal (Fe^3+^) charge transfer is observed. 

It is worth mentioning that annealing of Zn*_y_*Fe_3−*y*_O_4_ nanoparticles can also have detrimental effects like the migration of zinc cations, which would lead to the formation of two different crystalline phases, stoichiometric ZnFe_2_O_4_ and hematite [45]. Moreover, Zn*_y_*Fe_3−*y*_O_4_ (0.01 ≤ *y* ≤ 0.81) nanoparticles (3–11 nm) prepared by microwave refluxing method without going through any annealing treatment exhibits physical properties like bulk Zn*_y_*Fe_3−*y*_O_4_. Besides, the controlled synthesis of these nanoparticles encounters various obstacles (such as large size distribution, surface segregation, and aggregation), which hinders much potential use in real-world applications.

### 3.2. Nanocrystalline Thin Films (1 nm < Grain Size < 100 nm)

Because physical properties strongly depend on the cation distribution in nanocrystalline ZnFe_2_O_4_ thin films, the growth of stoichiometric ZnFe_2_O_4_ thin films by physical vapor deposition (PVD) are crucial. In the literature, nanocrystalline ZnFe_2_O_4_ thin films have been grown by a range of deposition techniques, such as sputtering [19], molecular beam epitaxy (MBE) [46] and pulsed laser deposition (PLD) [39], and chemical methods [32]. In these methods, the low growth temperatures often produce disordered ZnFe_2_O_4_ because it involves quenching of randomly distributed Zn^+2^ and Fe^+3^ vapor phases. 

**Figure 6:**
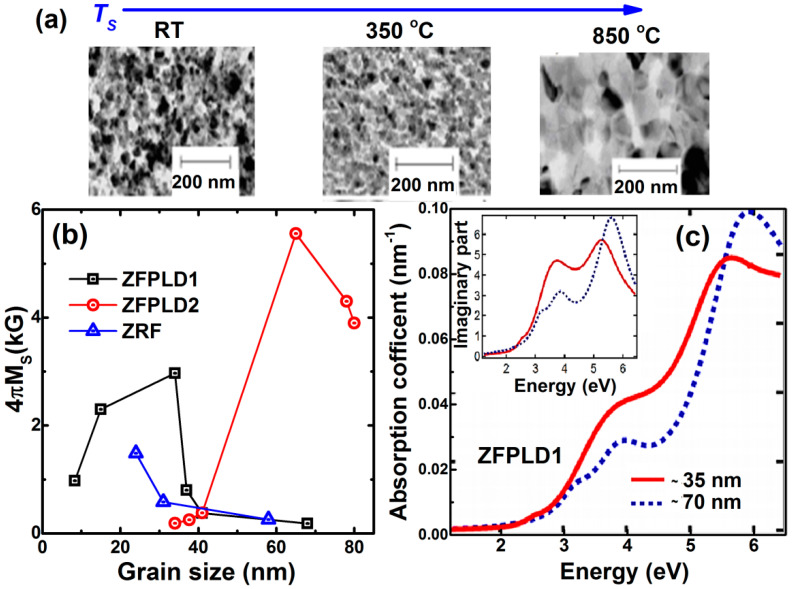
(**a**) TEM images of ZFPLD1 films grown at *T_S_* of RT, 350 °C and 850 °C exhibit their nanocrystalline nature (**b**) Room-temperature spontaneous magnetization (4πMs) values vs. grain sizes in ZFPLD1, ZFPLD2, and ZFRF films (Reproduced with permission from [39]. Copyright AIP Publishing, 2006). (**c**) Absorption coefficient of ZFPLD1 films with grain size of ~35 nm (full line) and ~70 nm (dashed line). Corresponding imaginary part of permittivity are plotted in the Inset (Reproduced with permission from [14]. Copyright AIP Publishing, 2015).

Previously, Bohra et al. [39] deposited ZnFe_2_O_4_ thin films under two different growth conditions: (i) in oxygen partial pressure of 0.16 mbar (ZFPLD1) and (ii) in the vacuum of 1 × 10^−5^ mbar (ZFPLD2) at in-situ growth temperatures, *T_S_* varies from room temperature (RT) to 850 °C from the ZnFe_2_O_4_ target on amorphous quartz substrate. The nanocrystalline nature of representative ZFPLD1 films with increasing grain sizes 10–70 nm can be seen in Figure 6a. The room temperature magnetic ordering has been observed in low grain-sized ZFPLD1 and ZFPLD2 films as shown in Figure 6b, indicating partial cation inversion. However, this structure goes back to the normal bulk spinel structure in higher grain sized films, which is paramagnetic. This feature is further confirmed in radio frequency (RF)-sputtered ZnFe_2_O_4_ films (ZFRF). Interestingly, we can see that even though the same grain-sized ZnFe_2_O_4_ films synthesized by different growth conditions show different magnetic properties and corresponding cation inversion.

Besides, ZFPLD1 films show typical insulator behavior with low oxygen vacancies as can be seen in spectroscopic ellipsometry given in Figure 6c [14], where the absorption edge is situated at the photon energy, *E* = 2.5 eV, hinting presence of only Fe^+3^ ions. The imaginary part of permittivity, *Im*{ε_0_} in the Inset, confirms the partial transfer of *Fe*_3þ_ cations from octahedral to tetrahedral sites compared to higher grain-sized films. The peak centered near 3.7 eV becomes enhanced, while the peak centered near 5.6 eV is reduced and shifted to lower *E*. Likewise, in-situ *T_S_*, an ex-situ annealing temperature (*T_A_*) and film thickness also plays a significant role in controlling cation inversion [47]. The ferrimagnetic ordering develops in nano thick and low *T_A_* annealed ZnFe_2_O_4_ films [47]. There have been also reports on the growth of nanocrystalline Zn*_y_*Fe_3−*y*_O_4_ films (*y* = 0, <1) with enhanced resistivity values by 10^2^–10^3^ orders, this might be attributed to the presence of large grain boundary volumes [46].

These nanocrystalline ZnFe_2_O_4_ films create a highly porous morphology which may offer a large number of electrochemically active sites, facilitates Li^+^ insertion/extraction inducing the improvement of rate capability and cycling stability [48]. The large surface area and suitable porosity further enhance specific capacitance which allows short diffusion channels for ions to migrate to the interior surface of the electrode and result in an enhanced current response [25]. 

### 3.3. Epitaxial Films (1 nm < Nano-Thick < 200 nm)

For the efficacious development of ZnFe_2_O_4_ thin film-based devices, the inherent characteristics of nanocrystalline thin films, such as grain boundary volume, the presence of defects/vacancies, pinholes, and internal stresses can be minimized by growing epitaxial ZnFe_2_O_4_ thin films. Besides, various technological applications require (i) inverted ferrimagnetic spinel structure [49,50] and (ii) the materials to be semiconducting and preferably transparent [49]. Various groups have attempted to grow epitaxial ZnFe_2_O_4_ thin films on single-crystal substrates [50,51]. To fabricate semiconducting Zn*_y_*Fe_3−*y*_O_4_ thin films, the depositions are carried out in reducing atmosphere, which causes partial transformation of Fe^+3^ into Fe^+2^ state at octahedral B sites. 

**Figure 7:**
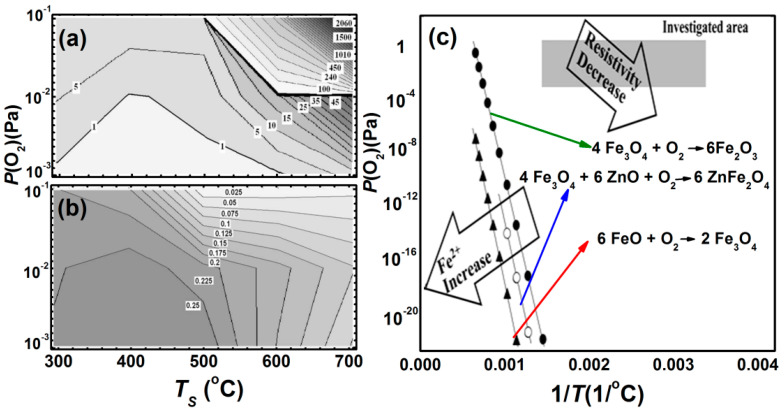
(**a**) Variation of room temperature resistivity (Ω-cm) and (**b**) saturated magnetization (emu/mm^3^) of Zn_y_Fe_3−y_O_4_ thin films with oxygen pressure, *P*(O_2_) and substrate temperature, *T*_S_, respectively. (**c**) Comparison between thermodynamic equilibrium lines (the amount of Fe^2+^) and resistivity variation trend (Reproduced with permission from [44]. Copyright AIP Publishing, 2007).

**Figure 8:**
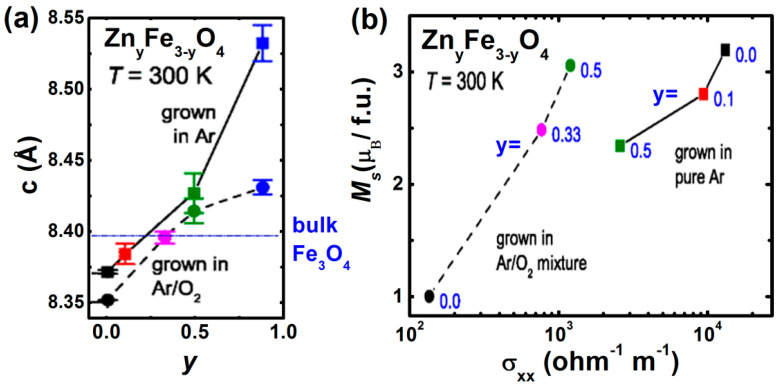
(**a**) Change in the *c*-axis lattice parameter of Zn_y_Fe_3__−y_O_4_ thin film grown in pure Ar atmosphere (squares) and an Ar/O_2_ mixture (circles) with Zn content *y*. (**b**) Correlation between saturation magnetization *M*_S_ and conductivity σ_xx_ (Reproduced with permission from [46]. Copyright American Physical Society, 2009).

Marcu, et al. [44] have investigated, in detail, the room temperature electric transport properties of ZnFe_2_O_4−δ_ thin films grown by PLD under varying oxygen pressure *P*(O_2_) and substrate temperature *T_S_*. It can be seen that decreasing both *T_S_* and *P*(O_2_) result in a decrease in the film resistivity (Figure 7a). The saturated magnetization *M_S_* increases with decreasing both *T_S_* and *P*(O_2_) (Figure 7b). Apart from the role of Fe^2+^ ion concentration, due to oxygen vacancies, the transport properties are also strongly influenced by structural disorders and vacancies. They also constructed a growth phase diagram about the stability of ZnFe_2_O_4−δ_ thin films and their possible dissociation into the solid solution of Fe_3_O_4_ and ZnO at higher *T_S_* and *P*(O_2_) (Figure 7c). Ferrimagnetic Zn*_y_*Fe_3−*y*_O_4_ (0 ≤ *y* ≤ 0.9) thin films were grown by Venkateshvaran, et al., both in pure Ar atmosphere and in Ar/O_2_ mixture, using laser MBE [46]. These films exhibit lattice parameters, slightly larger than bulk Zn*_y_*Fe_3__−*y*_O_4_ with increasing Zn content (*y)*, as shown in Figure 8a [17]. This feature has been ascribed to the epitaxial strain and larger radius of Zn^2+^(0.6 Å) compared to the Fe^3^ (0.49 Å). The electrical conductivity (σ) and the saturation magnetization (*M*_S_) show a correlation (Figure 8b) and any spin canting on the B sublattice reduces the *M*_S_, which also results in a reduction of σ, because the hopping amplitude is significantly suppressed if spin magnetic moments of atoms in neighboring B sites are not parallel. This result indicates that epitaxial thin films have less grain boundary volumes, so that itinerant electrons bring ideal and strong double exchange-like interactions between Fe ions at the B site. In particular, the epitaxial Zn*_y_*Fe_3__−*y*_O_4_ film is a suitable system to achieve physical properties that are theoretically designed. 

### 3.4. Other Nanostructured ZnFe_2_O_4_ Geometries

**Figure 9:**
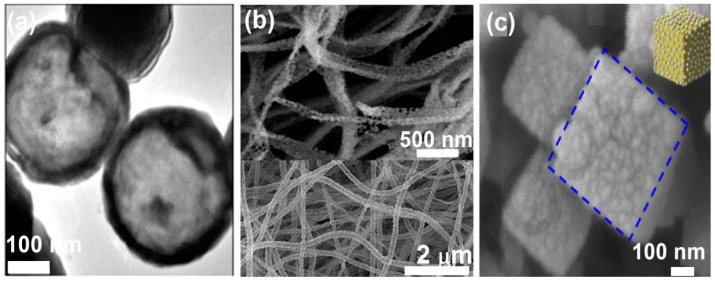
(**a**) High-resolution TEM of Zn_0_._3_Fe_2_._7_O_4_ nanospheres (Reproduced with permission from [52]. Copyright American Physical Society, 2019). (**b**) SEM images of nanotubes in the lower panel and SEM images of nanobelt in the upper panel (Reproduced with permission from [58]. Copyright Elsevier, 2018). (**c**) SEM images of hollow porous core-shell ZnFe_2_O_4_/AgCl nanocube (blue dotted line represents cubic facet) coated with EDTA-Ag nanoparticles (Reproduced with permission from [59]. Copyright Elsevier, 2020).

Whilst, quantum amount of work has been done in thin-film form, little is known about the potential use of ZnFe_2_O_4_ for device applications in nanoparticle morphology, as a result, an alternate approach is to tailor the shape of the particles since anisotropy plays a crucial role in deciding many surface-enhanced physical properties. Recently, Saha et al. synthesized [52] nano hollow spheres (NHSs) (shown in Figure 9a) instead of nanoparticles of Zn_y_Fe_3__−y_O_4_ by template-free solvothermal method, which shows an increase in *M*_S_ values with Zn doping, attaining a maximum at *x* = 0.2 (*M*s = 92.52 emu/g at 300 K), similarly to the bulk Zn_y_Fe_3__−y_O_4_. Therefore, enhanced magnetism with a decrease in conductivity, permittivity, and dipolar interaction enables Zn_y_Fe_3__−y_O_4_ NHSs to be a useful material for high-frequency applications [39,53,54,55,56,57]. 

Porous ZnFe_2_O_4_ nanotubes (Figure 9b) have been fabricated by electrospinning followed by two-step calcination in the atmosphere [58]. When the calcination temperature was increased from 600 °C to 650 °C, the ZnFe_2_O_4_ nanotubes evolved into well-crystalline nanobelts (Inset Figure 9b) due to the faster gas diffusion, more active grain growth and atomic diffusion rate caused by the relatively high temperature. The control experiments indicated the small addition of ZnFe_2_O_4_ can greatly enhance the photocatalytic activity. The hollow porous core-shell ZnFe_2_O_4_/AgCl nanocubic coated with EDTA (Ethylenediaminetetraacetic acid)–Ag nanoparticles [59] synthesized via a hydrothermal route followed by a self-etching process can be used as visible-light-triggered antibacterial agent (see Figure 9c). The hollow porous cores not only enhance the reflection and scattering of visible light but also facilitate the transfer rate of photogenerated electrons. These porous nanostructures of ZnFe_2_O_4_ can display potential practical applications.

## 4. Applications

These aforementioned tunable behaviors of nanostructured ZnFe_2_O_4_ have recently been found to have many technological applications in magnetic data storage, microwave components and energy conversion, and storage devices. Besides, they are fundamentally attractive to understand the structure–property correlation. 

### 4.1. Exchange Coupling

#### 4.1.1. Exchange Spring System (Soft + Hard Ferrite)

**Figure 10:**
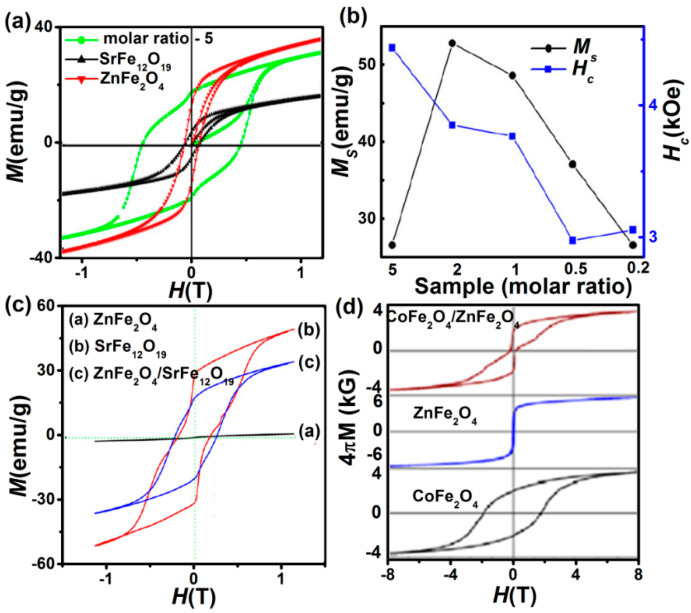
(**a**) *M-H* loops of SrFe_12_O_19_/ZnFe_2_O_4_ with molar ratio 5 (Reproduced with permission from [61]. Copyright Elsevier, 2018). (**b**) Variation of *Ms* and *Hc* values with molar ratio changing from 5 to 0.2. (**c**) *M*-*H* loops of SrFe_12_O_19_/ZnFe_2_O_4_ with normal structured ZnFe_2_O_4_ (Reproduced with permission from [62]. Copyright Elsevier, 2013). (**d**) *M-H* loops of exchange-coupled CoFe_2_O_4_/ZnFe_2_O_4_ bilayer at 10 K (Reproduced with permission from [60]. Copyright AIP Publishing, 2013).

The composite materials containing hard and soft magnetic materials, which are sufficiently exchange-coupled, can be recognized as an exchange spring magnet [60]. The merging of the high coercive field (*H*_C_) of the hard phase and large saturation magnetization (*M*_S_) of the soft phase can enhance magnetic properties of the permanent magnets [61]. Soft-magnetic inverted ZnFe_2_O_4_ nanostructure has several merits, such as high chemical stability and corrosion resistivity, superior magnetic properties, and low cost [61,62]. Thus, combining soft phase ZnFe_2_O_4_ along with hard phase SrFe_12_O_19_ has stimulated the researcher’s interest recently in high-performance nanocomposite magnets. Figure 10a shows room temperature exchange coupling property in SrFe_12_O_19_/ZnFe_2_O_4_ composites synthesized by coprecipitation method [61] with larger *M*_S_ and *H*c values as compared to the pure SrFe_12_O_19_. The molar ratio of SrFe_12_O_19_ influences the magnetic properties of SrFe_12_O_19_/ZnFe_2_O_4_ composites (Figure 10b). On the other hand, using normal spinel ZnFe_2_O_4_ in these composites [62] yields *M*_S_, and *H*_C_ of 35 emu/g and 2254 G (Figure 10c), respectively, indicating the composite has a greater capacity to avoid demagnetization. Nanocrystalline CoFe_2_O_4_/ZnFe_2_O_4_ bilayers (Figure 10d) also exhibit significant exchange coupling at low temperatures 10 K, which also retains up to room temperature for specific growth conditions [60]. 

#### 4.1.2. Exchange Bias (AFM/FM Interfaces)

**Figure 11:**
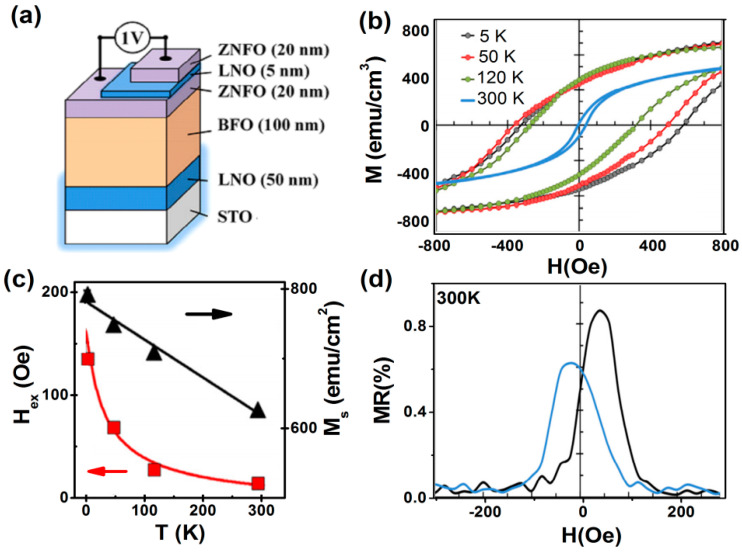
(**a**) Schematic of spin valve with the ferroelectric anti-ferromagnet BFO as the pinning layer and the proposed materials for other epitaxial layers. (**b**) M-H loops recorded upon heating up. The sample was annealed at *H*_ann_ = 3 kOe from 400 °C to room temperature before the measurements. The arrow indicates the direction of *H*_ann_. (**c**) Temperature dependence of the exchange bias (*H*_ex_) and saturation magnetization (*M*s). (**d**) The *MR* measured in such a spin valve heterostructures (Reproduced with permission from [64]. Copyright Elsevier, 2013).

The exchange bias (EB) is the magnetic interface effect that couples an antiferromagnetic (AFM) and a ferromagnetic (FM) system [63]. It manifests itself as a shift *H*_EB_ of the *M*–*H* loop along the magnetic field (*H*) axis and as an enhancement of the coercive field, *H*_C_, when the system is cooled down in an external magnetic field through the magnetic ordering temperatures of the AFM (*T*_N_) and FM (*T*_C_) phases. Exchange bias is one of the key concepts in spin valves, which has revolutionized the field of magnetic recording and memory devices, by allowing the pinning of the magnetization direction of one of the magnetic electrodes. Lin, et al. [64] fabricated an all-oxide spin valve with the ferroelectric antiferromagnet BiFeO_3_ (BFO) as the pinning AFM-layer (*T*_N_ = 385 °C). The multi-layered spin-valve, where two ferrimagnetic (FM) Zn_0_._7_Ni_0_._3_Fe_2_O_4_ (ZNFO) layers are separated by a nonmagnetic conducting layer, was grown epitaxially on a (001) SrTiO_3_ substrate, as shown in Figure 11a. They discussed some of the key physical and material issues for building up such novel devices in particular the hetero-epitaxy-induced strain effects on the electrical and magnetic properties of each layer and the establishment of exchange bias between BFO and ZNFO. The spin-valve was field annealed from a temperature above the high Néel point of BFO, after which a very large exchange bias field (*H*_ex_) was achieved at 5 K (Figure 11b) and kept at a decent value at room temperature (Figure 11c). The magnetoresistance (*MR*) achieved at room temperature (Figure 11d) was magnetically tunable in a similar way to conventional metallic spin valves. 

### 4.2. High-Frequency Applications

Performance of on-chip X-band (8–12 GHz) inductor, integrated with magnetic film can enhance its inductance density as well as the quality factor, facilitating miniaturization of RF devices and reducing dependency on silicon [54,55,56]. To find a suitable candidate material, whose ferromagnetic resonance frequency (*f*_FMR_) is above 6 GHz, i.e., in the expected new spectrum for 5G mobile communication, is now indispensable. Among metallic alloys, amorphous films, granular films and soft-magnetic ferrites, the first three exhibits very high permeability that results in >20% enhancement of inductance, but their applicability is limited to a few GHz; eddy-current losses and ferromagnetic resonance losses become prohibitively large at higher frequencies [55]. However, the family of ferrites with very high electrical resistivity and high *f*_FMR_ values can limit the aforesaid losses up to a few tens of GHz.

**Figure 12:**
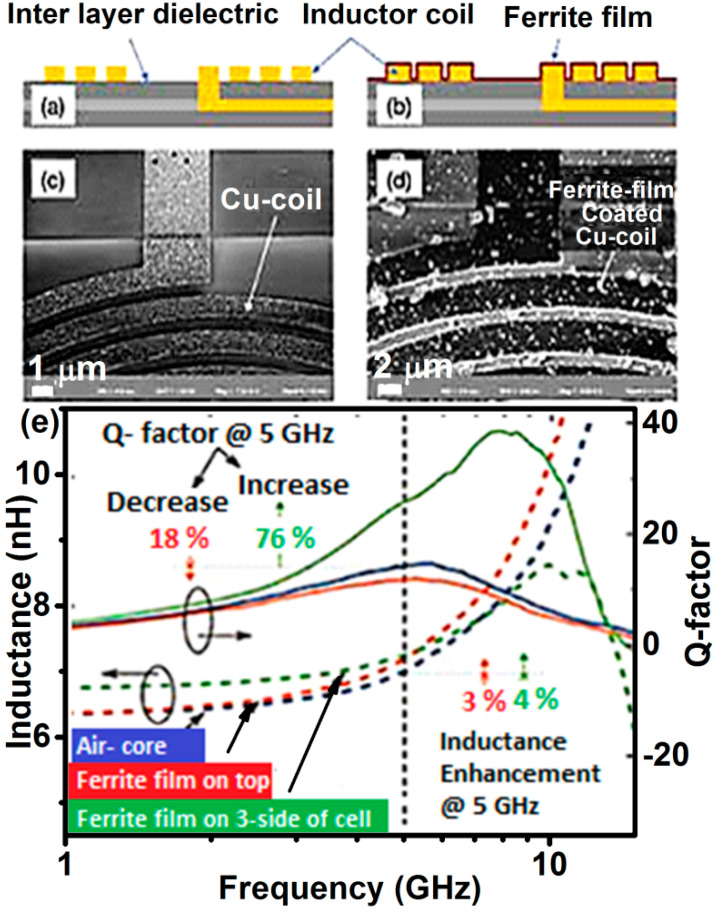
Schematic of inductor coil on a Si-CMOS chip (**a**) with passivation removed from 3-sides of the coil, and (**b**) with ZnFe_2_O_4_ thin (250 nm) film-coated conformally; (**c**,**d**) show the SEM images of the coil before and after ferrite coating; (**e**) Measured inductance and Q-factor of the on-chip inductance with ferrite film deposited only on top (in red) and surrounding 3-sides of the coil (in green) (Reproduced with permission from [55]. Electrochemical Society, 2017).

A crucial challenge is the growth of thick ferrite films on a silicon chip in a CMOS (complementary metal oxide semiconductor)-compatible manner [55]. Most PVD methods are either non-scalable or require high processing temperatures (in-situ or ex-situ annealing ≥ 500 °C). On the other hand, the low-temperature chemical methods require strict control of the *pH* of the solution, which may otherwise corrode the on-chip metal wiring. Recently, Sai et al. deposited partially inverted ZnFe_2_O_4_ film with soft magnetic characteristics (*M*_S_ = 130 emu/cc and *H*_C_ = 120 Oe) directly on a Si-CMOS integrated circuit by Microwave-Assisted Synthesis Technique (MAST) at 200 °C [54,55]. These films showed FMR frequency above 30 GHz, with negligible FMR loss below 15 GHz, therefore, they could be used as inductor core in the frequency range up to 15 GHz and as an electromagnetic noise suppressor around 30 GHz. Up to 13% enhancement in inductance density and 25% enhancement in the quality factor were demonstrated at 10 GHz, giving the highest-density (450 nH/mm^2^) on-chip ferrite-core inductor. To harness the best effect of magnetic film, a complete magnetic path, i.e., complete encapsulation of the coil, is necessary. By utilizing the ability of MAST to deposit ZnFe_2_O_4_ film conformally, three sides of the on-chip coil are covered by ZnFe_2_O_4_ film in a single step. The resulting coil structure is demonstrated both schematically and with SEM images in Figure 12a–d. An enhancement of the Q-factor by 78% is achieved, as shown in Figure 12e. It is to be noted that the magnetic path is, nevertheless, not closed. A very large increase in inductance and inductance density can be achieved if the coil can be fabricated on a ferrite layer instead of the interlayer dielectric. An important aspect of the development of RF-CMOS integrated circuits is the design and fabrication of the magnetic-core inductor at low temperature and scalable level, for this purpose, RF sputtered inverted ZnFe_2_O_4_ films of narrow FMR line width of 40 Oe (at 9 GHz) [53] could then be explored to meet the ever-increasing demand for functionality.

### 4.3. Lithium-Ion Batteries

Global Lithium-ion battery (LIBs) deployments stand poised to grow substantially for electric vehicles and renewable energy storage in the coming years, but it will be necessary to search/design novel electrode materials. There have been many reports on the ZnFe_2_O_4_ as anode materials for LIBs owing to their high electrochemical properties (high specific capacity, cycling performance, rate capability, and reversible specific capacity). Instead, traditional graphite anodes exhibit theoretical specific capacity only 372 mAhg^−1^ [65,66], limited energy density, and poor electrochemical performance, unsatisfying the demand of many practical applications. The Li-ion storage mechanism of ZnFe_2_O_4_ involves conversion and alloying reaction, where each unit of ZnFe_2_O_4_ has been reported to be able to store up to 9 units of Li^+^ ions, thus giving it a high theoretical capacity of 1072 mAhg^−1^ [24]. Compared to other spinel transition-metal oxides, ZnFe_2_O_4_ possess the advantage to be non-toxic and less expensive than *M*Co_2_O_4_ compounds, while Mn-based spinel oxides display lower electrical conductivity. Magnetite, Fe_3_O_4_, has a theoretical capacity of 900 mAhg^−1^, but it displays a high working potential of 2.1 V vs. Li^+^/Li, limiting thus the energy storage capability [67,68]. It has been evidenced that Zn-doping of Fe_3_O_4_, with carbon coating, can enhance the electrochemical performance by increasing the electronic and ionic conductivity and could work for relatively low voltage [69]. 

Prior studies have shown that reducing particle size can help to relax the strain, and have a high surface-to-volume ratio and reduced transport length, which can lead to increased cyclability. Based on the Li storage mechanism [24,70,71] of ZnFe_2_O_4_, the first discharge cycle is described as follows:

ZnFe_2_O_4_ + 0.5Li^+^ + 0.5e^−^ → Li_0_._5_ZnFe_2_O_4_ (1)

Li_0_._5_ZnFe_2_O_4_ + 1.5Li^+^ + 1.5e^−^ → Li_2_ZnFe_2_O_4_ (2)

Li_2_ZnFe_2_O_4_ + 6Li^+^ + 6e^−^ → 4Li_2_O + Zn + 2Fe (3)

Zn + Li^+^ + e^−^ ↔ LiZn (alloy) (4)

In recharging process, the ferrite molecule cannot be recovered and the reactions involve the newly formed oxides ZnO and Fe_2_O_3_ [24,72,73]:

3Li_2_O + 2Fe ↔ Fe_2_O_3_ + 6Li^+^ + 6e^−^ (5)

Li_2_O + Zn ↔ ZnO + 2Li^+^ + 2e^−^ (6)

**Figure 13:**
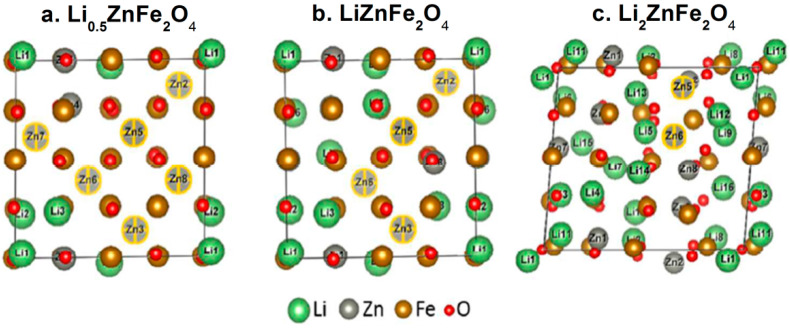
Structures of (**a**) Li_0_._5_ZnFe_2_O_4_, (**b**) LiZnFe_2_O_4_, and (**c**) Li_2_ZnFe_2_O_4_. (grey with a yellow circle: Zn^+2^ ions in 8a site) (Reproduced with permission from [74]. Copyright American Chemical Society, 2017).

It is interesting to note that the formation of the LiZn alloy has recently been debated as experimental measurements associated with DFT calculations have found no evidence of it and suggested the formation of FeO instead of Fe_2_O_3_ (Figure 13) [74,75]. This study also shed light on the structure of Li_x_ZnFe_2_O_4_, in which Li atoms are first inserted in the vacant *16c*Wyckoff sites (for 0 < *x* ≤ 1). When increasing the Li content (*x* > 0.25), some migration of Zn^2+^ cations from tetrahedral *8a* sites to vacant *16c* octahedral sites also occur; when no vacant 16 sites are left, remaining Li atoms will take place in *8a* sites (1 < *x* ≤ 2). 

**Figure 14:**
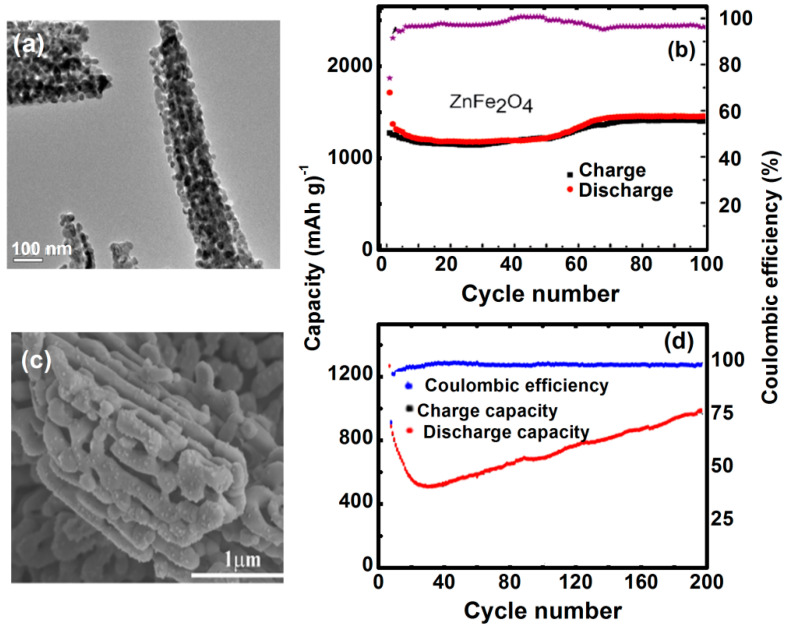
(**a**) TEM image of ZnFe_2_O_4_. (**b**) Cycle performances and Coulombic efficiencies (CE) of ZnFe_2_O_4_ at a current density of 100 mA g^−1^ (Reproduced with permission from [77]. Copyright Royal Society of Chemistry, 2015). and (**c**) SEM image of ZFPE-15 and (**d**) cycle performances and Coulombic efficiencies (CE) at a current density of 1A g^−1^ (Reproduced with permission from [86]. Copyright Elsevier, 2020).

The porous structures have attracted significant attention, due to their high surface area and buffer effects, which are preferable for improving the electrochemical properties [76]. However porous framework can not only accommodate the volume expansion/contraction when reacting with Li^+^, but also provide more reaction sites on the surface and shorten the diffusion distance of Li^+^ and electrons. Hou, et al. reported the porous ZnFe_2_O_4_ inflorescence spicate structure assembled by spherical nanoparticles as primary building particles (Figure 14a), which is synthesized by the precipitation method and subsequent thermal treatment by using cetyltrimethylammonium bromide (CTAB) as a surfactant [77]. The reversible capacity for spicate ZnFe_2_O_4_ remains 1398 mAhg^−1^ over 100 cycles, which is higher than that of reported different morphologies of pure ZnFe_2_O_4_ electrodes (Table 1). The cyclic performances and Coulombic efficiencies for ZnFe_2_O_4_ at a high current density 100 mAg^−1^ are illustrated in Figure 14b. The discharge capacity of the ZnFe_2_O_4_ electrode is much higher than its theoretical capacity, which is due to the high active surface and interface area of the porous nanostructures. 

**Table 1:** Morphology dependent electrochemical performance of ZnFe_2_O_4_ as electrode.

Morphology	Reversible Capacity mAh g^−1^	Cycle	Current Rate mA g^−1^	Ref.
Thin film	434	100	10	[78]
Nanoparticles	841	50	60	[79]
Nanofibers	733	30	60	[80]
Nano-octahedrons	910	80	60	[81]
Nanorod	900	50	100	[82]
Cubic nanoparticles	367	50	60	[83]
Hollow spheres	900	50	65	[70]
Hollow microspheres	1200	120	100	[84]
Hollow nanospheres	1101	120	200	[85]

Moreover, the ZnFe_2_O_4_ composite displays electrochemical properties. For instance, the conducting polymer poly(3,4-ethylene dioxythiophene) (PEDOT) coated ZnFe_2_O_4_ composites (Figure 14c) delivered a discharge capacity of 1510.5 mAhg^−1^ at 100 mA g^−1^ after 200 cycles, exhibiting the high performance over others and were much larger than that of pure ZnFe_2_O_4_. The high-rate cycling performance and corresponding Coulombic efficiencies of the ZFPE-15 electrode were tested at a large current density of 1 A g^−1^, as shown in Figure 14d. The electrically conductive PEDOT coating facilitates electron transfer from ZnFe_2_O_4_ and acts as a buffer matrix to restrain volume expansion, showing that ZnFe_2_O_4–_15 wt% PEDOT composites (ZFPE-15) are promising anode materials for use in LIBs [86]. Hence developments in LIBs provide new insights about the processes ruling their fundamental chemical properties and this should inspire more efforts in developing low-cost ZnFe_2_O_4_ based electrodes for LIBs, with enhanced rate capability and cycling life. 

### 4.4. Photoelectrochemical (PEC) Water Splitting

Among the various paths for solar fuel production, the photochemical dissociation of water into its constituent parts, H_2_ and O_2_ offer the simplest and potentially efficient approach which requires virtually zero energy input except sunlight to produce clean and storable hydrogen as a fuel. Many photocatalytic anode materials suffer from poor light absorption at visible wavelengths, poor charge transport, and/or poor photo-stability in aqueous electrolyte solutions. A theoretical solar-to-hydrogen (STH) conversion efficiency close to 20% was predicted for the n-type narrow bandgap (*E*_g_ = 1.9 eV) ZnFe_2_O_4_ semiconductor. A sufficient positive valence band of ZnFe_2_O_4_ can drive PEC water-splitting when used as an anode material [16,87]. 

**Figure 15:**
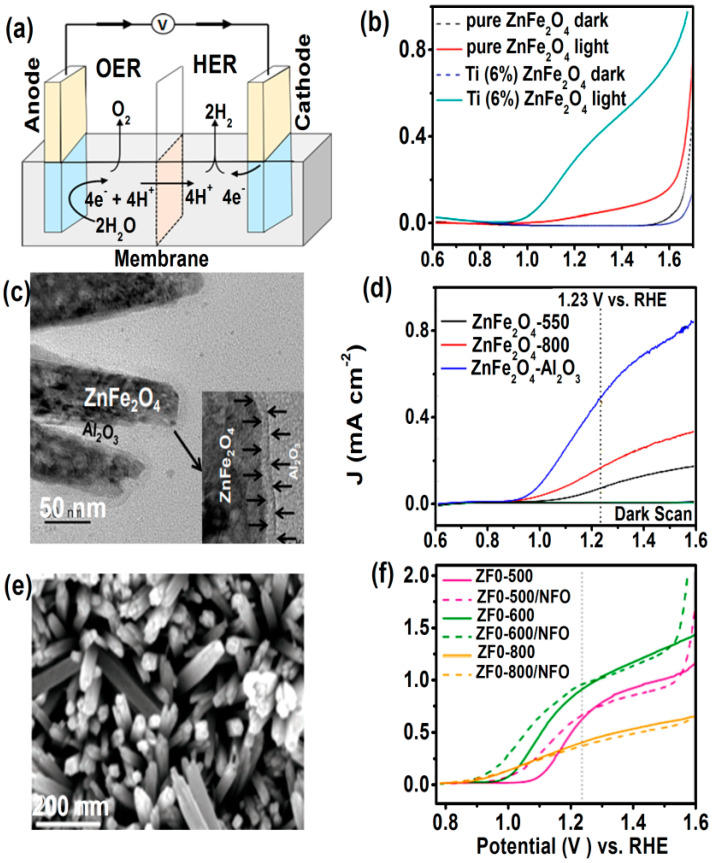
(**a**) Schematic of electrolysis cell for water splitting, (**b**) current density J vs. potential curves, V of ZnFe_2_O_4_ photoanodes (Reproduced with permission from [92]. Copyright Royal Society of Chemistry, 2017). (**c**,**d**) TEM image of ZnFe_2_O_4_-Al_2_O_3_ and corresponding J vs. V curves (Reproduced with permission from [93]. Copyright Royal Society of Chemistry, 2018). (**e**) SEM images ZnFe_2_O_4_ nanorod, ZFO-500 (**f**) J vs. V curves (Reproduced with permission from [95]. Copyright Wiley, 2018).

The electrochemical cell under basic conditions (see Figure 15a) can undergo redox reaction, like hydrogen evolution reaction (HER) and oxygen evolution reaction (OER), which can be expressed as:

4OH^−^ → O_2_ + 2H_2_O + 4e^−^ (OER at the anode)

4H_2_O + 4e^−^ → 2H_2_ + 4OH^−^ (HER at the cathode)

However, different treatments on the ZnFe_2_O_4_ electrode shows enhancement in water splitting photocurrent density (*J*) such as, post-synthesis with hydrogenation (at mild temperature) enhances electrical conductivity by introducing oxygen vacancies [88]. Hybrid microwave annealing treatment is better than conventional annealing treatment [89,90], and doping of semiconductor (e.g., p-type Co-ZnFe_2_O_4_) [91] leads to change in electrical conductivity and offers high crystallinity. 

Guo, et al. [92] synthesized Ti^4+^ doped ZnFe_2_O_4_ as anode material by effective spray pyrolysis method for water splitting application. The substitution of Fe^3+^ by Ti^4+^ enhances the charge carrier concentration and electron transfer efficiency. Ti-doped ZnFe_2_O_4_ photoanodes exhibit, *J* = 0.35 mA cm^−2^ at 1.23 V vs. RHE (reversible hydrogen electrode), which is 8.75 times higher than that of the pure ZnFe_2_O_4_ photoanodes, as shown in Figure 15b [92]. 1-D ZnFe_2_O_4_ nanorods with Al_2_O_3_ passivation layer at different annealing temperatures showed an increased *J* value of 0.48 mA/cm^2^ at 1.23 V vs. RHE (Figure 15c,d) compared to seven times and three times higher than pure ZnFe_2_O_4_ annealed at 550 °C and 800 °C, respectively. High-temperature annealing and coating of an Al_2_O_3_ layer helped to minimize surface defects and reduced surface recombination due to the chemical passivation effect [93]. On the other hand, PEC performance of inverted ZnFe_2_O_4_ nanorod photoanode prepared by conversion route [94], shown in Figure 15e [95] strongly depends upon cation inversion (*x)*, which further depends upon growth temperatures. The *J*–*V* curves in Figure 15f for 600 °C grown sample (ZFO-600, *x* = 0.18) delivers the highest *J* surpassing 0.8 mA cm^−2^ at 1.23 V and rising to 1.7 mA cm^−2^ at 1.6 V versus RHE. While ZFO-800, *x* = 0.13 sample exhibited the most favorable photocurrent onset potential (at ≈0.8 V vs. RHE), *J* remained below 1.0 mA cm^−2^. By contrast, the ZFO-500, *x* = 0.3 sample exhibited a more positive onset potential (≈1.2 V vs. RHE) but *J* was higher than ZFO-800 at the high applied potential. The NiFe_2_O_4_ (NFO) coated ZnFe_2_O_4_ nanorods show higher performance as photoanodes. The higher *x* is related to the superior charge transport and changes in *x* result in changes in the electronic structure. However, still, *J* is far below the theoretical maximum value of ≈11 mA cm^−2^ with ZFO which has to increase by overcoming fundamental limitations, such as poor absorption coefficient and bulk charge separation, for enhancement of PEC water splitting performance. 

### 4.5. Electrochemical Supercapacitors

**Figure 16:**
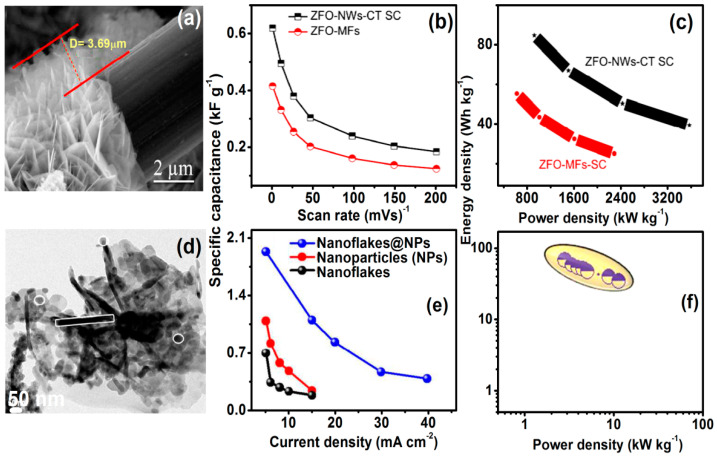
Hydrothermal method: (**a**) SEM images of ZFO precursor nanowall arrays on CT fibres, (**b**) specific capacitances as a function of scan rate, and (**c**) comparative Ragone plots (Reproduced with permission from [98]. Copyright Elsevier, 2019). In-situ bio-mediated green rotational chemical bath deposition: (**d**) TEM of ZnFe_2_O_4_ nano-flakes@ZnFe_2_O_4_/C nanoparticle thin film heterostructure, (**e**) plot of specific capacitance (F g^−1^) vs. current density (mA cm^−2^) and (**f**) Ragone plot (Reproduced with permission from [99]. Copyright American Chemical Society, 2017).

Electrochemical supercapacitors are energy storage devices with properties intermediate to those of batteries and electrostatic capacitors. They exhibit high power density (ten times higher than batteries), high cycling stability, high energy capacity, storage for a shorter period, and high charging/discharging, which makes them a contender for next-generation power devices [4]. ZnFe_2_O_4_ is a suitable material for electrochemical applications, due to its eco-friendly nature, sufficient resources, cost-effectiveness, strong redox process, and an extraordinary theoretical capacity of 2600 F g^−1^ [23,96]. However, its lower conductivity and low cycling stability make it unsuitable for efficient supercapacitors. To overcome these issues, conducting materials were added to the ZnFe_2_O_4_ to enhance the electronic conductivity and cycling stability [97]. Javed, et al. fabricated flexible supercapacitors with ZnFe_2_O_4_ nanowall (NWs) arrays deposited on carbon textile (ZFO-NWs-CT) electrode by hydrothermal method, as shown in Figure 16a. These supercapacitors exhibit capacitance of 620 F g^−1^ at 5 mVs^−1^ compared to the pure ZnFe_2_O_4_ NWs (Figure 16b) with a long life of 10,000 cycles. The Ragone plot in Figure 16c of ZFO-NWs-CT supercapacitors shows a high energy density of 85 Wh kg^−1^ at a power density of 1000 W kg^−1^ [98]. 

Vadiyar, et al. [99] synthesized composite of ZnFe_2_O_4_ nano-flakes and carbon nanoparticles are shown in Figure 16d by in-situ bio-mediated green rotational chemical bath deposition, which demonstrates specific capacitance of 1884 F g^−1^ at a current density of 5 mA cm^−2^ (Figure 16e) and energy density of 81 Wh kg^−1^ at a power density of 3.9 kW kg^−1^, as shown in Figure 16f. This supercapacitor also exhibits long cycle stability of 35,000 cycles by losing only 2% capacitance, which is attributed to the self-assembled organization of the heterostructures with the addition of carbon to ZnFe_2_O_4_ [99]. Recently, a nanocomposite of ZnFe_2_O_4_ nanorods and reduced graphene oxide showed higher specific capacitance 1419 F/g with cyclic stability of 93% after 5000 cycles at the scan rate of 10 mV/s. Thus, ZnFe_2_O_4_/carbon hybrid materials are promising electrode material for supercapacitor. 

## 5. Conclusions

Current day research aims at revolutionizing energy storage devices with advanced materials that can operate at low power consumption with high speed, yet without compromising the aim of shrinking their size. The search of more efficient materials should not be done at the expense of the environment or health preservation. For this goal, materials such as ZnFe_2_O_4_ possess many advantages, owing to its physico-chemical properties, rich phase diagram with multiple conductive or magnetic states which are dictated by its complex and open atomic structure. The presented comprehensive review on the growth methodologies of various ZnFe_2_O_4_ nanostructures, (nanoparticles and epi/poly thin films *etc*.) and its famed cation inversion engineering not only portrays the current knowledge about possible changes brought forth in structural/chemical, electronic, and magnetic properties, but also helps to envision the future research directions to develop ZnFe_2_O_4_ towards efficient energy material.

Moreover, its spinel structure being relatively “open”, many vacant crystallographic sites can facilitate the intercalation of (mobile) dopants, which in turn can enlarge the number of applications of such material. Further, as summarized in this review, different growth conditions can be used to control and tune the magnitude of defects and grain boundaries, off stoichiometry, non-zero Fe^2+^/Fe^3+^ ratios, and micro/nano strains, which ultimately allows designing the overall properties to enhance the energy-efficiency of ZnFe_2_O_4_ material. Considering nanoscale cation engineering and with the achievable control over growth strategies of various ZnFe_2_O_4_ nanostructures, ZnFe_2_O_4_ can be a potential material in the following futuristic applications:

- Different spintronics devices, possibly with low-energy operation cost, can be constructed by using an inverted stoichiometric ZnFe_2_O_4_ thin film as ferrimagnetic layer in magnetic tunnel junctions, as a barrier layer in spin filtering devices, oxygen-deficient Zn*_y_*Fe_3−*y*_O_4−δ_ thin film as a conducting layer could be used in homo-epitaxial devices, provided with a fine control of the stoichiometry during the growth.
- Inverted ZnFe_2_O_4_ thin layer with low microwave loss can be a potential material for high-frequency applications, such as 5G mobile communication.
- Inverted ZnFe_2_O_4_ nanostructures are emerging photoanode material for photoelectrochemical solar fuel productions. Cation disorder in ZnFe_2_O_4_ facilitates photogenerated charge separation and increased charge carrier transport.
- ZnFe_2_O_4_ used as an electrode in a Li-ion battery demonstrated large charge/discharge capacity and cycle stability. Highly porous surface and wide voids in ZnFe_2_O_4_ nanostructures play a critical role in enhancing electrochemical reactions. The suitable cathode and stable electrolyte materials are the prerequisite to form ZnFe_2_O_4_-based Li-ion battery considering high working voltage of electrode.
- Various ZnFe_2_O_4_-based heterostructures and nanocomposites with high conducting property can boost cycle stability and energy density for high-performance supercapacitors.

Convincingly, the cation inversion and various porous nanostructures are important factors to tailor the properties of ZnFe_2_O_4_ which can potentially lead into useful nanoscale devices, although scalable energy-efficient devices using ZnFe_2_O_4_ at nanoscale remains to be major challenge to date, and may require significant advancements in research and development efforts combined with fundamental research on ZnFe_2_O_4_.

## Data Availability

No new data were created or analyzed in this study. Data sharing is not applicable to this article.
